# A Genome-Wide Association Study Reveals Genes Associated with Fusarium Ear Rot Resistance in a Maize Core Diversity Panel

**DOI:** 10.1534/g3.113.007328

**Published:** 2013-11-01

**Authors:** Charles T. Zila, L. Fernando Samayoa, Rogelio Santiago, Ana Butrón, James B. Holland

**Affiliations:** * Department of Crop Science, North Carolina State University, Raleigh, North Carolina 27695; † Misión Biológica de Galicia, CSIC, Pontevedra, Spain, 36080; ‡ U.S. Department of Agriculture—Agricultural Research Service, Plant Science Research Unit, Raleigh, North Carolina 27695

**Keywords:** association analysis, disease resistance, genotype-by-environment interaction, maize, quantitative trait

## Abstract

*Fusarium* ear rot is a common disease of maize that affects food and feed quality globally. Resistance to the disease is highly quantitative, and maize breeders have difficulty incorporating polygenic resistance alleles from unadapted donor sources into elite breeding populations without having a negative impact on agronomic performance. Identification of specific allele variants contributing to improved resistance may be useful to breeders by allowing selection of resistance alleles in coupling phase linkage with favorable agronomic characteristics. We report the results of a genome-wide association study to detect allele variants associated with increased resistance to *Fusarium* ear rot in a maize core diversity panel of 267 inbred lines evaluated in two sets of environments. We performed association tests with 47,445 single-nucleotide polymorphisms (SNPs) while controlling for background genomic relationships with a mixed model and identified three marker loci significantly associated with disease resistance in at least one subset of environments. Each associated SNP locus had relatively small additive effects on disease resistance (±1.1% on a 0–100% scale), but nevertheless were associated with 3 to 12% of the genotypic variation within or across environment subsets. Two of three identified SNPs colocalized with genes that have been implicated with programmed cell death. An analysis of associated allele frequencies within the major maize subpopulations revealed enrichment for resistance alleles in the tropical/subtropical and popcorn subpopulations compared with other temperate breeding pools.

The hemibiotrophic fungus *Fusarium verticillioides* (Sacc) Nirenberg is endemic in most maize fields in the United States and is present in many arable regions of the world (Van Egmond *et al.* 2007). This fungus causes *Fusarium* ear rot disease of maize, especially in low-rainfall, high-humidity environments, such as the southern United States and some lowland tropics (Miller and Trenholm 1994). Infection by *F. verticillioides* can result in decreased grain yields, poor grain quality, and contamination by the mycotoxin fumonisin, a suspected carcinogen associated with various diseases in livestock and humans (Miller and Trenholm 1994; Marasas 1996; Presello *et al.* 2008).

The best strategy for controlling *Fusarium* ear rot and reducing the incidence of fumonisin contamination of grain is the development and deployment of maize hybrids with genetic resistance. *Fusarium* ear rot resistance is under polygenic control and strongly influenced by environmental factors; no fully immune genotypes have been discovered (King and Scott 1981; Nankam and Pataky 1996; Clements *et al.* 2004). The complexity of this resistance trait has impeded breeding, such that most commercial maize hybrids have lower levels of resistance than are desirable (Bush *et al.* 2004). Linkage-based mapping studies in biparental populations have shown that *Fusarium* ear rot resistance quantitative trait loci (QTL) have relatively small effects and are not consistent between populations (Pérez-Brito *et al.* 2001; Robertson-Hoyt *et al.* 2006; Ding *et al.* 2008; Mesterházy *et al.* 2012).

Despite the genetic complexity of resistance to *Fusarium* ear rot and fumonisin accumulation and despite the very low heritability of resistance measured on individual plants, resistance on the basis of family means from well-replicated studies is moderately to highly heritable (Robertson *et al.* 2006; Eller *et al.* 2008; Bolduan *et al.* 2009). Robertson *et al.* (2006) and Bolduan *et al.* (2009) reported genotypic correlations between ear rot resistance and fumonisin accumulation of 0.87 in North Carolina and 0.92 in Germany, respectively, indicating that visual selection on *Fusarium* ear rot resistance should be effective in simultaneously reducing fumonisin contamination. The heritability estimates predict, and empirical selection studies demonstrate, that selection for improved ear rot resistance can be effective (Robertson *et al.* 2006; Bolduan *et al.* 2009; Eller *et al.* 2010). Unfortunately, most sources having high levels of ear rot resistance are older or exotic unadapted inbreds that lack the agronomic performance of modern elite maize lines (Clements *et al.* 2004; Eller *et al.* 2008, 2010). Thus, breeders are faced with the difficulty of introducing polygenic resistance alleles of generally small effect linked to inferior polygenic alleles for agronomic performance if they attempt to incorporate improved genetic resistance from unadapted lines into elite breeding gene pools. Identification of specific allelic variants that confer improved resistance would permit maize breeders to select for rare recombinant chromosomes in backcross progeny that have desired target resistance allele sequences in coupling phase with the favorable elite polygenic background, facilitating the improvement of disease resistance without decreasing agronomic performance.

Resolving small-effect QTL to causal genes for traits that are difficult to accurately measure phenotypically is exceedingly difficult in biparental mapping populations (Holland 2007). Compared with traditional linkage-based analyses, association mapping offers greater mapping resolution while eliminating the time and cost associated with developing synthetic mapping populations (Flint-Garcia *et al.* 2005; Yu and Buckler 2006). Historically, a major limitation to association mapping in low linkage disequilibrium (LD) species such as maize has been the large number of genetic markers required to detect marker-trait associations. Limiting the search space to predetermined candidate genes allows for association mapping with a smaller number of markers but requires extensive knowledge of the biochemical pathway contributing to the trait of interest (Remington and Purugganan 2003). To date, nothing is known about the pathways contributing to *Fusarium* ear rot resistance in maize. However, the recent availability of the maize 50k SNP genotyping array (Ganal *et al.* 2011) has provided almost 50,000 SNP markers scored on 279 of the 302 inbred lines of a commonly used maize core diversity panel (Flint-Garcia *et al.* 2005; Cook *et al.* 2012). The maize diversity panel captures much of the diversity present in public breeding programs worldwide. The large number of markers available on the diversity panel has enabled genome-wide association studies (GWAS) for several complex traits in maize, including kernel composition traits (Cook *et al.* 2012) and the hypersensitive response (Olukolu *et al.* 2013). Olukolu *et al.* (2013) identified SNPs associated with the hypersensitive defense response in or adjacent to five genes not previously known *a priori* to affect disease resistance but whose predicted gene functions all involved the programmed cell death pathway. In this study, we used GWAS to identify SNPs associated with *Fusarium* ear rot resistance in the maize core diversity panel both within and across two contrasting environments: North Carolina, United States, and Galicia, Spain.

## Materials and Methods

### Genotypes and experimental design

The maize core diversity panel [sometimes referred to as the “Goodman” association panel, because the seed stocks were originally assembled by Major Goodman at North Carolina State University (Flint-Garcia *et al.* 2005)] was evaluated in several years in both North Carolina, United States, and Galicia, Spain. Only the 279 inbred lines with available genotypic data were considered in this study. In the Galicia experiment, a subset of 270 inbred lines from the maize diversity panel was evaluated for *Fusarium* ear rot resistance in a randomized 15 × 18 *α*-lattice block design with two replicates in 2010 and 2011. Nine lines with insufficient seed were dropped from the Galicia experiment before randomization. In the North Carolina experiment, the maize diversity panel was part of an evaluation of the entire U.S. Department of Agriculture maize seed bank collection of inbred lines in 2010 (Romay *et al.* 2013) and subsets of that collection evaluated in 2011 and 2012. The genotypic data on the maize seed bank collection reported by Romay *et al.* (2013) were not available at the time of analysis. The 2010 seed bank collection evaluation included 2572 inbred line entries and was arranged in an augmented, single-replicate design. Experimental entries were divided into 18 sets of differing sizes on the basis of maturity and field assignment. Each block within each set was augmented with a B73 check plot in a random assignment, and five other checks (IL14H, Ki11, P39, SA24, and Tx303) were included once per set in a random position. Romay *et al.* (2013) reported flowering time evaluations of the entire collection evaluated at three locations in 2010, including North Carolina. Here we include data only from North Carolina because it was the only environment used for *Fusarium* ear rot evaluation.

In 2011 and 2012, the maize core diversity panel was part of a larger sample of inbreds evaluated. The larger population consisted of 771 diverse entries divided into eight sets on the basis of maturity and replicated across years. Although disease measurements were collected on all experimental entries in both years, genotypic data were not available on inbreds outside of the core diversity panel at the time of analysis. Sets were randomized within the field, and each set was blocked using an *α*-lattice design. As with the seed bank collection evaluation, each block was augmented by a randomly assigned B73 check plot, and five other checks (GE440, NC358, NK794, PHB47, and Tx303) were included once per set.

The three North Carolina environments were artificially inoculated with local toxigenic *Fusarium verticillioides* isolates via the toothpick method (Clements *et al.* 2003). Approximately 1 wk after flowering, a toothpick containing *F. verticillioides* spores was inserted directly into the primary ear of five plants in each plot. At maturity, inoculated ears were harvested and visually scored for *Fusarium* ear rot symptoms. Scores were assigned to each ear in increments of 5% from 0% to 100% diseased based on the percentage of the ear presenting disease symptoms (Robertson *et al.* 2006; Supporting Information, Figure S1). In Galicia, between 7 and 14 d after flowering, five primary ears per plot were inoculated with 2 mL of a spore suspension of the local toxigenic isolate of *F. verticillioides*. The spore suspension contained 10^6^ spores mL^−1^ and was prepared following the protocol established by Reid *et al.* (1996) with some modifications. Inoculum was injected into the center of the ear with a four-needle vaccinator that perforated the husks and injured three to four kernels. Ears from each plot were collected 2 mo after inoculation and were individually rated for *Fusarium* ear rot symptoms by the use of a seven-point scale (1 = no visible disease symptoms, 2 = 1–3%, 3 = 4–10%, 4 = 11–25%, 5 = 26–50%, 6 = 51–75%, and 7 = 76–100% of kernels exhibiting visual symptoms of infection, respectively) devised by Reid and Zhu (2005). Phenotypic data on the seven-point scale from the Galicia environments were transformed to the 0–100% scale used in North Carolina in the analyses. Reliable data could not be obtained for some line-environment combinations because seed set for some plots was limited as the result of poor adaptation. Raw data are provided in File S1. Climate data from on-farm weather stations were obtained from http://www.nc-climate.ncsu.edu/ and http://www2.meteogalicia.es/galego/observacion/estacions/listaEstacions.asp.

### Genotypic data

The genotypic data were 47,445 SNPs from the Illumina maize 50k genotyping array filtered by Olukolu *et al.* (2013). The original array consists of 49,585 SNPs designed by Ganal *et al.* (2011). Olukolu *et al.* (2013) filtered the data set to include only those SNP markers that mapped to defined single locations in the maize genome and had <20% missing data (http://www.genetics.org/content/suppl/2012/12/05/genetics.112.147595.DC1/genetics.112.147595-3.txt).

### Statistical analyses

#### Estimation of least-square means and heritabilities:

The Galicia and North Carolina experiments were analyzed separately and then combined in a single multienvironment analysis. Each year of data within each experiment was first analyzed separately by fitting a mixed linear model that included line as a fixed effect, silking date as a fixed linear covariate, and replication (Galicia only), block within replication (Galicia only), set (North Carolina only), and block within set (North Carolina only) as random effects. The mixed linear model for the Galicia experiment across years included line as a fixed effect, silking date as a fixed linear covariate, and year, line × year interaction, replication within year, and block within replication as random effects. The North Carolina experiment was analyzed across years with a model including line as a fixed effect, silking date as a fixed linear covariate, and year, line × year interaction, set within year, and block within set as random effects. In the combined experiment analysis, each combination of location and year was considered an environment. The combined analysis model included a fixed line effect, silking date as a fixed covariate nested within environment, a random line × environment interaction effect, and nested random experimental design effects (replication within environment and block within replication at Galicia and set within environment and block within set at North Carolina). All analyses were weighted by the number of ears scored within each plot and used a heterogeneous error variance structure. In both experiments, larger predicted ear rot values were associated with larger residuals, so a natural logarithmic transformation of raw ear rot scores (which largely eliminated the relationship between residual variance and predicted values) was used for all analyses. Least square means were estimated for 267 inbred lines within each experiment and across experiments (File S2) with the use of ASReml version 3 software (Gilmour *et al.* 2009). Means for 12 lines were not estimable due to missing phenotypic observations in all environments (generally because of poor seed production).

We conducted a second analysis treating inbred lines as random effects for the purposes of estimating heritability for *Fusarium* ear rot resistance in the diversity panel. The same models as above were used except lines were treated as random effects to obtain estimates of genetic variance. Line mean-basis heritability was estimated as

$${\hat{H}}_{c}=1-\frac{\sigma_{PPE}^{2}}{2{\hat{\sigma }}_{G}^{2}}$$

where $\sigma_{PPE}^{2}$ is the average prediction error variance for all pairwise comparisons of lines and ${\hat{\sigma }}_{G}^{2}$ is the estimated genetic variance (Cullis *et al.* 2006). We estimated line mean-basis heritabilities for each environment individually, across the North Carolina environments, across the Galicia environments, and we also estimated line mean-basis heritability for the combined data set across all environments. The model used to estimate line mean-basis heritability in the combined data set was further modified by nesting the random line effect within environment and modeling the genotype-by-environment effect (**G**) matrix as unstructured, thereby allowing estimation of unique genetic variance within each environment and a unique genetic correlation between each pair of environments. For the purpose of estimating heritability, the average of the 10 pair-wise covariance estimates between environments (which are expected to equal the genotypic variance) was used in the denominator of the above equation.

Silking date heritabilities also were calculated for each environment and across environments. The same models used to compute ear rot heritabilities were used to estimate silking date heritabilites, but silking date was treated as the dependent variable instead of as a fixed linear covariate.

### Association analyses

A genetic kinship matrix (**K**; File S3) based on observed allele frequencies (VanRaden 2008; method 1) was created using R software version 3.0.0 (R Core Team 2013). A subset of 4000 SNP markers were used to estimate **K**. Markers were uniformly distributed across the genome (at least 60 kbp between adjacent markers) and had no missing data after we excluded heterozygous genotypes. Olukolu *et al.* (2013) used a kinship matrix produced by Tassel software (Bradbury *et al.* 2007), which is appropriate for population structure correction for GWAS. In addition to population structure correction, we also wanted to estimate the polygenic background genetic variance component, so we estimated a new **K** matrix that is scaled appropriately to represent realized genomic average identity by descent relationships among the lines (VanRaden 2008).

Tassel version 4.1.24 was used for the GWAS based on a mixed linear model (Bradbury *et al.* 2007). The least-square means for inbred lines were used as the input phenotypes, and each set of means (North Carolina, Galicia, and combined) was analyzed separately (File S2). The mixed linear model implemented by Tassel was

y=Xβ+Zu+e

where ***y*** is the vector of ear rot least square means (on the natural-log scale), ***β*** is a vector of fixed effects, including SNP marker effects, ***u*** is a vector of random additive genetic effects from background QTL for lines, ***X*** and ***Z*** are design matrices, and ***e*** is a vector of random residuals. The variance of the ***u*** vector was modeled as

$$\text{Var}\left(\text{u}\right)=\text{K}\sigma_{\text{a}}^{2}$$

where **K** is the *n* × *n* matrix of pairwise kinship coefficients ranging 0−2 and $\sigma_{\text{a}}^{2}$ is the estimated additive genetic variance (Yu *et al.* 2006).

Restricted maximum likelihood estimates of variance components were obtained by use of the optimum compression level and population parameters previously determined options in Tassel (Zhang *et al.* 2010). The optimum compression level option reduces the dimensionality of **K** by clustering *n* lines into *s* groups on the basis of their genomic similarity, thereby reducing computational time and potentially improving model fit. The *P*-values for each of the 47,445 tests of associations between one SNP and ear rot resistance within each analysis were used to estimate the false-positive discovery rate using the QVALUE version 1.0 package in R (Storey and Tibshirani 2003). SNPs significant at *q* < 0.10 in the initial GWAS scan for a particular environment set were then included together in a joint SNP association model together using the GLM procedure in SAS software version 9.2 (SAS Institute Inc 2010) to estimate the total amount of variation explained by the SNPs together and to re-estimate their effects jointly. Candidate genes either containing or located adjacent to associated SNPs were identified using the MaizeGDB genome browser (Andorf *et al.* 2010).

### Allele frequency analysis

Lines were grouped into one of five major maize subpopulations (stiff stalk [SS], non-stiff stalk [NSS], tropical/subtropical [TS], popcorn [PC], and sweet corn [SC]) on the basis of the population structure analysis of the maize core diversity panel reported by Flint-Garcia *et al.* (2005) (http://panzea.org/db/gateway?file_id=pop_structure_xls). Lines of mixed ancestry (the result of admixture among the subpopulations) were dropped from the analysis. On the basis of the results of the association analyses, the frequencies of alleles that reduced disease severity at significant SNPs were estimated within each subpopulation using the FREQ procedure using SAS software version 9.2 (SAS Institute Inc 2010). At each SNP locus, a Fisher’s exact test in R software version 3.0.0 (R Core Team 2013) was used to test the null hypothesis that frequency of the allele conferring increased disease resistance was the same across all five subpopulations.

## Results

### Line means and heritability

Significant (*P* < 0.001) genotypic variation for ear rot resistance was observed in both the North Carolina and Galicia experiments. The mean ear rot observed among 267 inbred lines of the association panel ranged from 4.4 to 100% with an overall mean of 41.1% in North Carolina and from 0 to 89.3% with an overall mean of 7.4% in Galicia (File S2 and Table S1). In the combined analysis, mean ear rot ranged from 1.6 to 79.6% with an overall mean of 22.1%. The silking date covariate was highly heritable (${\hat{H}}_{c}$ = 0.98 in the combined analysis) and was significantly associated with ear rot resistance in the North Carolina and combined analyses (*P* < 0.001), but not in the Galicia analysis (*P* = 0.099, Table S1).

A significant (*P* < 0.001) line × environment interaction was detected in the combined analysis. Results of the mixed model analysis that estimate unique genotypic covariances for each pair of environments indicated that the two Galicia environments had a much stronger genotypic correlation (*r* = 0.93; Table 1 and Figure S2) than did any other pair of environments (range, *r* = 0.28 to 0.51; Table 1 and Figure S2). Thus, there was little genotype-by-environment (G × E) interaction between the two Galicia environments, and the heritability of line means across the 2 yr in Galicia was 0.71. In contrast, pair-wise genotypic correlations were much lower among the North Carolina environments and between North Carolina and Galicia environments (Table 1 and Figure S2), generating much of the observed G × E interaction in the combined analysis. Despite the strong G × E interaction among North Carolina environments, heritability of genotype means across the 3 yr in North Carolina (0.73) was greater than within any single North Carolina environment (Table S1). In addition, heritability of line means across all five environments was 0.75, greater than within any single environment or group of environments (Table S1). Therefore, we conducted separate association analyses on three different sets of genotypic mean values for ear rot: (1) means from three North Carolina environments, (2) means from two Galicia environments, and (3) means from the combined analysis of all five environments.

**Table 1:** Genotypic covariance/variance/correlation matrix for *Fusarium* ear rot from the combined analysis of a maize diversity panel evaluated in five environments

Environment	NC 2010	NC 2011	NC 2012	Galicia 2010	Galicia 2011
NC 2010	**0.27**	0.42	0.44	0.51	0.44
NC 2011	0.15	**0.45**	0.38	0.33	0.28
NC 2012	0.19	0.21	**0.68**	0.36	0.35
Galicia 2010	0.15	0.12	0.17	**0.32**	0.93
Galicia 2011	0.11	0.09	0.14	0.25	**0.23**

The diagonal (bold) is an estimate of genetic variance (${\hat{\sigma }}_{G}^{2}$) plus the genotype-by-environment interaction (${\hat{\sigma }}_{GE}^{2}$) within each environment. Estimates of genetic variance (covariance between pairs of environments) are shown below the diagonal, and genetic correlations between inbred lines in each pair of environments are shown above the diagonal. NC, North Carolina.

### Association mapping of *Fusarium* ear rot resistance

**Figure 1:**
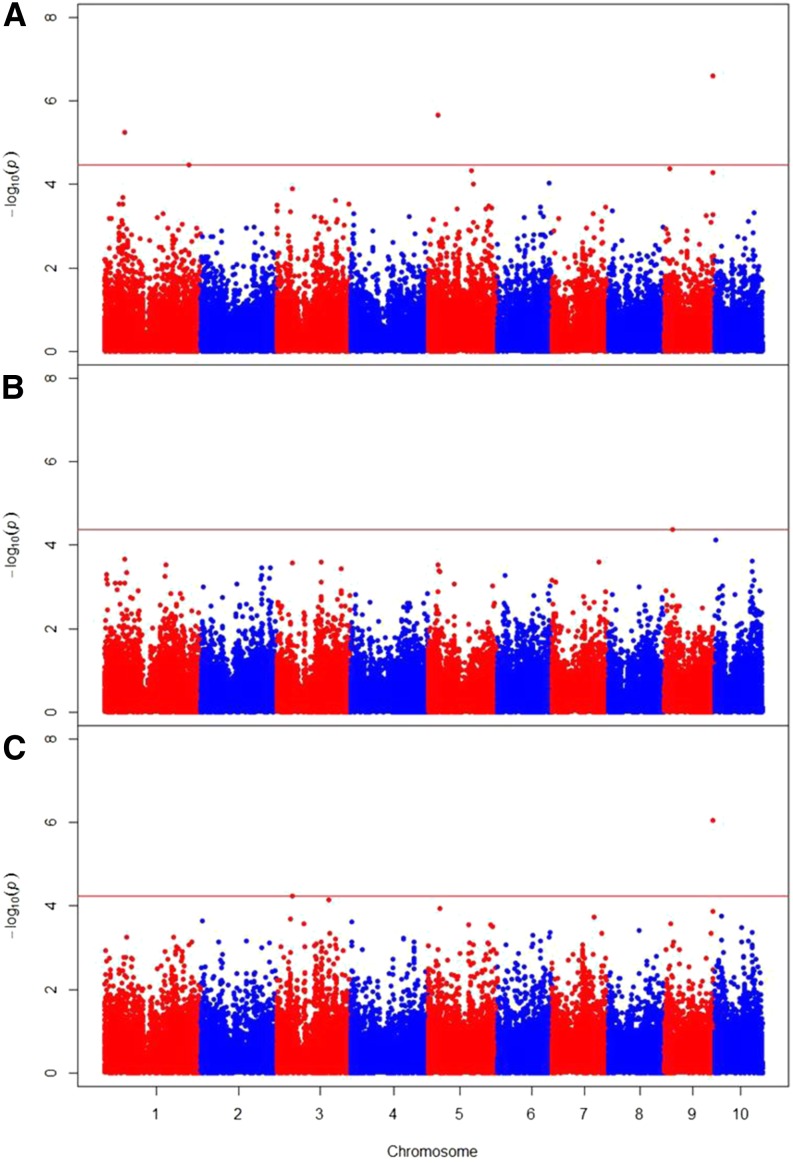
Results of the three GWAS showing significant associations (points above red false-positive discovery rate = 0.10 threshold lines) in the North Carolina (A), Galicia (B), and combined (C) analyses. The vertical axis indicates –log_10_ of *P*-value scores, and the horizontal axis indicates chromosomes and physical positions of SNPs.

The optimum compression option in Tassel clustered the 267 lines into 229 groups in the Galicia analysis and 197 groups in the North Carolina and combined analyses (Table 2). Background genetic effects modeled by **K** accounted for 31% of the total variation among line means in the North Carolina analysis, 57% of the total phenotypic variation in the Galicia analysis, and 48% of the total phenotypic variation in the combined analysis (Table 2). In the analysis of means from North Carolina environments, two SNPs were identified as significantly associated with ear rot resistance at *q* ≤ 0.05 (raw *P*-value = 2.4 × 10^−7^), and one additional SNP was identified at *q* ≤ 0.10 (Table 3 and Figure 1). In the combined analysis, one SNP was identified as significantly associated with ear rot resistance at *q* ≤ 0.05 and coincided with one of the SNPs identified in the North Carolina analysis. No SNPs significant at *q* ≤ 0.10 were identified in the Galicia analysis, where the minimum raw *P*-value among SNP association tests was 2.1 × 10^−4^.

**Table 2:** Number of lines, number of groups, compression level, polygenic additive background genetic variance component, residual genotypic variance component, and proportion of total line mean variance explained by additive relationship matrix from the three MLM analyses

	N*^a^*	Groups*^b^*	Compression*^c^*	(${\hat{\sigma }}_{G}^{2}$)*^d^*	(${\hat{\sigma }}^{2}$)*^d^*	$\left(\frac{{\hat{\sigma }}_{G}^{2}}{{\hat{\sigma }}_{G}^{2}+{\hat{\sigma }}^{2}}\right)$*^e^*
North Carolina	247	197	1.25	0.09	0.20	0.31
Galicia	254	229	1.11	0.18	0.14	0.57
Combined	267	197	1.36	0.10	0.11	0.48

MLM, mixed linear model; SNP, single-nucleotide polymorphism.

a Total number of lines included in the analysis.

b Number of groups determined by optimum compression.

c Compression level is the average number of individuals per group.

d Polygenic additive background genetic variance and residual genotypic variance components are estimated in Tassel by fitting the kinship matrix (**K**) in the mixed linear model without any SNP marker effects.

e Background genetic variance divided by total phenotypic variance.

**Table 3:** Chromosome locations (AGP v2 coordinates), allele effect estimates, genes containing or adjacent to SNP, and other summary statistics for the three SNPs significantly associated with *Fusarium* ear rot resistance in the North Carolina analysis and the single SNP associated with resistance in the combined analysis

Chromosome	SNP Physical Position, bp	*P*-Value	*q*-Value	Allele	N*^a^*	Allele Effect, %*^b^*	Additive Variance Estimate*^c^*	*R*^2^*^d^*	Gene Containing or Adjacent to SNP
North Carolina analysis									
1	63,540,590	5.5 × 10^−6^	0.084	A	224	+0.945	0.036	8.8	GRMZM2G703598
				G	22	0.0			
5	30,997,717	2.2 × 10^−6^	0.050	G	225	+1.149	0.042	9.6	GRMZM2G111477
				A	19	0.0			
9	151,295,233	2.4 × 10^−7^	0.011	A	67	−0.365	0.041	11.5	GRMZM2G178880
				G	176	0.0			
Galicia analysis									
1	63,540,590	0.826^NS^	1.000	A	231	+0.035	9.55×10^−5^	1.9×10^−2^	GRMZM2G703598
				G	22	0.0			
5	30,997,717	0.918^NS^	1.000	G	228	−0.017	2.49×10^−5^	4.2×10^−3^	GRMZM2G111477
				A	23	0.0			
9	151,295,233	0.198^NS^	1.000	A	71	−0.115	0.003	0.7	GRMZM2G178880
				G	179	0.0			
Combined analysis									
1	63,540,590	4.5 × 10^−3^	0.689	A	244	+0.425	0.010	3.1	GRMZM2G703598
				G	22	0.0			
5	30,997,717	2.6 × 10^−3^	0.689	G	240	+0.428	0.011	3.5	GRMZM2G111477
				A	24	0.0			
9	151,295,233	9.1 × 10^−7^	0.042	A	74	−0.292	0.024	9.6	GRMZM2G178880
				G	189	0.0			

Statistics from environments in which the SNPs were not significantly associated with ear rot are also shown for comparison. SNP, single-nucleotide polymorphism.

a N, total number of lines with the specific SNP genotype.

b Allele effects are reported back-transformed to the original 0–100% disease severity scale.

c Additive variance for an inbred population was computed as two times the product of the separate allele frequencies times the genotypic value from Tassel squared using the formula 2*pqa*^2^ from Bernardo (2002).

d *R*^2^, proportion of total line mean variance explained by SNP as computed by Tassel.

### Candidate genes colocalized with associated SNPs

**Figure 2:**
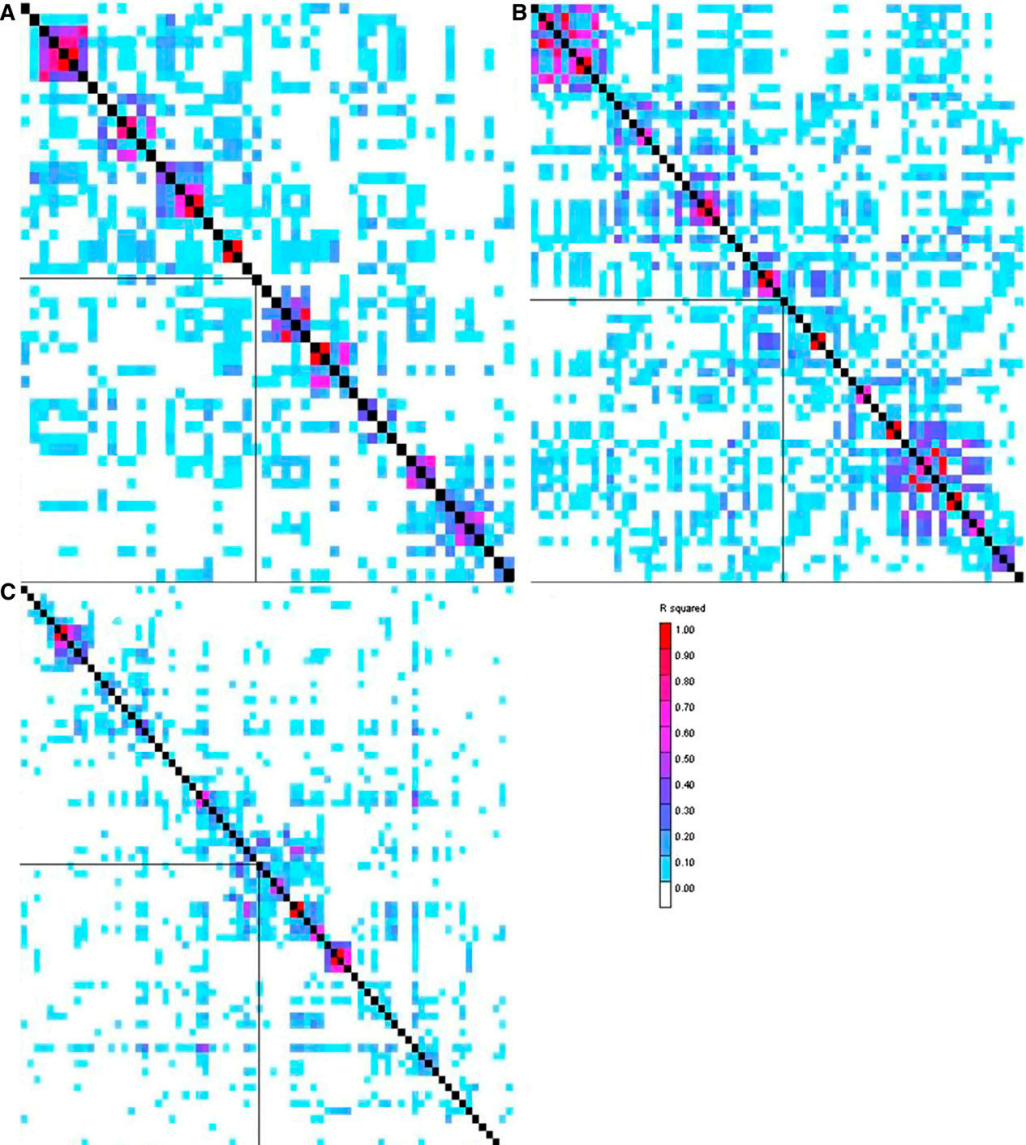
LD heatmaps showing LD measure (*r*^2^) calculated for each pairwise combination of SNPs in an approximately ±1 Mbp region surrounding each SNP significantly associated with ear rot resistance in the North Carolina analysis. (A) LD around chromosome 1 SNP. (B) LD around chromosome 5 SNP. (C) LD around chromosome 9 SNP. The significant SNP on each chromosome is highlighted by the perpendicular black lines within each heatmap. Colors indicate the magnitude of each pairwise *r*^2^ measure (*r*^2^ = 1 is red to *r*^2^ = 0 is white).

Genes containing or nearby SNPs significantly associated with ear rot resistance were characterized by the use of the filtered predicted gene set from the annotated B73 reference maize genome (Schnable *et al.* 2009). Two of the three genes identified in the North Carolina analysis have predicted functions that have been implicated in disease response pathways in other plant species (Tsunezuka *et al.* 2005; Hématy *et al.* 2009). The SNP at physical position 151,295,233 bp on chromosome 9, which was identified in both the North Carolina and combined analyses, is located in an intronic region of a cellulose synthase-like family A/mannan synthase gene (Table 3). Mean LD *r*^2^ between this SNP and other chromosome 9 SNPs dropped below 0.1 within approximately 100 kbp (Figure 2). The other two SNPs identified in the North Carolina analysis on chromosomes 1 and 5 were located inside of a gene of unknown function and nearby a heat-shock 60-kD protein (HSP60), respectively. Mean LD *r*^2^ between the chromosome 1 and chromosome 5 SNPs and other SNPs dropped below 0.1 within approximately 10 kbp and 100 kbp, respectively (Figure 2). Although the chromosome 1 and 9 SNPs were not significantly associated with ear rot resistance in Galicia, the allele effects at these loci were consistent between North Carolina and Galicia (Table 3). However, the allele effect at the chromosome 5 SNP locus showed a change in direction between North Carolina (+1.149%, Table 3) and Galicia (−0.017%).

### Allele frequencies at candidate genes

We estimated the allele frequency at the three SNPs significantly associated with ear rot resistance in five of the major maize subpopulations: SS, NSS, TS, PC, and SC (Flint-Garcia *et al.* 2005). European flint types are poorly represented in this maize core diversity panel and thus were not considered. Popcorn and sweet corn types were considered in the analysis, but comparisons with either of these two subpopulations may be less reliable than comparisons with other subpopulations because of smaller sample size (Table 4). The allele that reduced disease severity at the chromosome 1 SNP locus is only present in the NSS and TS subpopulations but not at high enough frequencies to be considered significantly different from the other three subpopulations (*P* = 0.15, Table 4). The allele with reduced disease severity at the chromosome 5 SNP locus is significantly (*P* = 6.2 × 10^−6^) over-represented in TS and PC lines relative to other temperate (SS, NSS, and SC) lines. At the chromosome 9 SNP locus, the allele associated with reduced disease severity is significantly (*P* = 3.846 × 10^−4^) overrepresented in PC lines compared with the other four subpopulations (Table 4). Averaging least square means from the combined analysis across members of each subpopulation, the SS, NSS, TS, PC, and SC subpopulations had average ear rot scores of 24.0%, 24.3%, 14.6%, 17.9%, and 46.5%, respectively (Table 4).

**Table 4:** Allele frequencies of significantly associated SNPs in the five major maize subpopulations

Chromosome	SNP Physical Position, bp	Allele Increasing Resistance	Allele Frequency, %						N					Ear Rot Mean, %*^a^*				
			SS	NSS	TS	PC	SC	*P*-value	SS	NSS	TS	PC	SC	SS	NSS	TS	PC	SC
1	63,540,590	G	0.0	8.4	15.4	0.0	0.0	0.1488	28	107	65	8	6	24.0	24.3	14.6	17.9	46.5
5	30,997,717	A	0.0	3.8	26.6	37.5	0.0	6.193 × 10^−6^	28	106	64	8	6					
9	151,295,233	A	14.3	34.9	26.6	100.0	33.3	3.846 × 10^−4^	28	106	64	7	6					

SNP, single-nucleotide polymorphism; N, total number of lines within each subpopulation; SS, stiff stalk; NSS, non-stiff stalk; TS, tropical/subtropical; PC, popcorn; SC, sweet corn.

a Overall phenotypic ear rot means are the average of least square means from the combined analysis across members of each subpopulation.

## Discussion

### Heritability and false discovery rate estimation

The mean ear rot severity observed across experimental entries was 41.1% in North Carolina and 7.4% in Galicia (Table S1). Mean ear rot in North Carolina 2012 was particularly high (55%; Table S1). The very strong genotypic correlation between Galician environments (Table 1 and Figure S2) justified their grouping as one environmental set in the analysis. Genotypic effects were significantly correlated between each pair of North Carolina environments, but at much lower magnitude (Table 1 and Figure S2). Genotypic values in North Carolina 2010 had slightly higher correlations with the genotypic values in Galicia than in other years of North Carolina (Table 1), so grouping the three North Carolina environments has little justification on the basis of G × E patterns. Nevertheless, this environment grouping has a natural interpretation in terms of geography and adaptation, and the heritability of line means across these environments was greater than any individual environment, such that analysis of the three North Carolina environments as a group simplified interpretation of results.

The relationship between the *F. verticillioides* isolates used in each location is unknown; as such, it is possible that differences in pathogen aggressiveness could have contributed to the disparity in mean ear rot values across environments. In addition, differences in inoculation methods, as well as variation in temperature and precipitation levels, may have allowed for more favorable disease development in North Carolina compared with Galicia. Although precipitation levels varied across all five environments, average daily temperatures (both pre- and postflowering) were greater in all three North Carolina environments compared with the two Galicia environments (Table S2).

Heritabilities observed across environments in this study (${\hat{H}}_{c}\geq 0.71$) are consistent with estimates from biparental populations (Robertson *et al.* 2006) and a small sample of North American and European public inbred lines (Bolduan *et al.* 2009). These heritability estimates were obtained with a model that assumed each line is a random sample from the reference population of global maize inbreds, modeled by a genotypic variance-covariance structure equal to the genotypic variance component multiplied by an identity matrix. For the purpose of controlling population structure in association analysis, adjusted line means from the original model were then used as observations in a mixed model analysis that modeled the genotypic variance-covariance structure as proportional to the realized genomic relationship matrix, thus incorporating the different pairwise relationships among the lines. This mixed model was simplified by the compression method of Zhang *et al.* (2010), which clusters lines according to genetic similarity and replaces the full pair-wise realized genomic relationship matrix with a reduced matrix of average relationships among the groups. The optimal level of clustering or compression is determined empirically based on model fit to the observed phenotypic data. A compressed relationship matrix can have better model fit than the original matrix when the empirically observed covariance relationships among lines follow the group relationship averages better than the individual pairwise relationships. Typically, this can happen when closely related lines are grouped and estimates of the group phenotypes and their relationships with other group phenotypes are improved. The optimal compression level can vary among phenotypes for the same set of lines, as observed in this study.

Among environment groups, the proportion of phenotypic variance explained by background genetic effects (**K**) was much smaller in North Carolina (31%, Table 2) compared with Galicia (57%). Besides the small polygenic additive effects captured by the kinship matrix, rare allele variants (minor allele frequency < 0.05) with larger effects, as well as epistatic interactions, may explain some of the genotypic variation not captured by either **K** or the significantly associated SNPs (Manolio *et al.* 2009).

Analyzing the Galicia environments separately from the North Carolina environments revealed no significant SNPs, whereas the North Carolina analysis identified three SNPs significantly associated with *Fusarium* ear rot resistance (Table 3). Examination of the empirical distribution of *P*-values for the Galicia analysis revealed a slight skew toward greater *P*-values, whereas the North Carolina and combined analyses exhibited excesses of small *P*-values (Figure S3, Figure S4, and Figure S5). The Storey and Tibshirani (2003) method used to compute the false-discovery rate assumes that the distribution of *P*-values for truly null tests follows a flat distribution, such that if the observed proportion of very low *P*-values is lower than expected based on the flat distribution, the false discovery rate will be high even for the lowest *P*-values, as we observed in the Galicia analysis. Whereas a few significant SNPs were identified in the North Carolina and combined analyses at *q* < 0.10, no SNP had a *q*-value of <0.9 in the Galicia analysis (Figure S3, Figure S4, and Figure S5). The disparity between the two individual experiment analyses highlights the importance of conducting individual environment association analyses in the presence of significant G × E interaction. It should be noted, however, that the appropriate threshold proportion of variation caused by G × E interaction to warrant individual location analyses instead of an overall combined analysis is not clear.

One possible mode of G×E interaction is the relative increase or decrease of additive allelic effects among different loci between environments (Falconer and Mackay 1996). Comparison of the absolute value of the allele effect at each of the identified SNP loci between North Carolina and Galicia revealed that allele effects were larger in North Carolina across all three loci (Table 3), congruent with the greater mean ear rot values in North Carolina (Table S1). The largest proportion of phenotypic variance explained by **K** was in Galicia (Table 2), and when combined with comparatively smaller allele effects, suggested that more loci may have contributed to ear rot resistance in Galicia than North Carolina, and on average each locus had a smaller additive effect on disease phenotype in Galicia. Collectively, these two points may explain the deficiency of SNPs significantly associated with ear rot resistance in the Galicia analysis.

### Association analyses

Three SNPs significantly associated with ear rot resistance were identified in the North Carolina analysis (Table 3), and all localized to separate chromosomes. One of these three SNPs, located on chromosome 9, was also identified in the combined analysis. None of the three SNPs localized to any of the linkage map bins containing resistance QTL reported by Robertson *et al.* (2006) and Ding *et al.* (2008). However, the proportion of phenotypic variance explained by each SNP is consistent with individual QTL *r*^2^ values reported by each of the two aforementioned mapping studies. The chromosome 9 SNP explained the largest proportion of the variation in line mean values for ear rot resistance (*R*^2^ = 11.5% in NC and *R*^2^ = 9.6% in the combined analysis, Table 3), whereas the chromosome 1 and chromosome 5 SNPs explained 8.8% and 9.6% of the variation in line mean values for ear rot resistance in North Carolina, respectively. Modeling all three SNPs together collectively explained 26% of the line mean variation in ear rot resistance in North Carolina.

Although all three SNPs explained a relatively large portion of the total variation in line means, each SNP had a relatively small additive effect on ear rot resistance (±1.1 percentage points ear rot severity on the back-transformed scale, Table 3). Additive genetic variance estimates for each SNP were computed based on allele effects and frequencies (Table 3), and when scaled to the total line mean variance coincided with the SNP *r*^2^ values computed by Tassel (Table 3). In every case, an increase in disease resistance (decrease in ear rot severity) was associated with the rare allele at each locus. Resistance alleles at the chromosome 1 and 5 SNP loci were overrepresented in the tropical subpopulation relative to the other temperate subpopulations (Table 3), consistent with enriched disease resistance observed in tropical maize for some foliar diseases of maize (Wisser *et al.* 2011; Olukolu *et al.* 2013) and the lower level of ear rot disease observed in tropical lines in this study.

Using the same association panel and marker set as this study, Olukolu *et al.* (2013) reported that LD in the maize core diversity panel is variable across chromosomes and subpopulations. The authors also reported that marker pairs separated by more than 10 kbp had *r*^2^ < 0.1 on average, which is consistent with estimates of *r*^2^ < 0.1 between marker pairs separated by 5−10 kbp on average in tropical subpopulations and 10−100 kbp on average in temperature subpopulations (Lu *et al.* 2011). Increased marker coverage, such as the genotype-by-sequencing data (Elshire *et al.* 2011) used in Romay *et al.* (2013), in conjunction with a larger association panel, may be able to uncover more SNPs in greater LD with ear rot resistance loci. Assuming an association panel of between 350 and 400 inbred lines, Van Inghelandt *et al.* (2011) indicated that as few as 4000 markers would be necessary in a GWAS to detect individual QTL explaining >10% of the total phenotypic variation for a complex trait within the stiff stalk subpopulation, whereas 65,000 markers would be required to detect QTL at the same threshold within European flint types. In a sample of 2815 inbred lines from the USA National Plant Germplasm System representing the same heterotic groups described in this study, Romay *et al.* (2013) reported that the use of more than 680,000 genotype-by-sequencing markers was sufficient to detect most known candidate genes associated with flowering time in maize. Even so, polymorphisms that strongly associated with the lower LD tropical/subtropical subpopulation (such as *ZmCTT*) were more difficult to detect compared with polymorphisms that more frequently associated with greater LD temperate subpopulations (such as *Vgt1*). The results of Romay *et al.* (2013) indicate that although increased marker coverage and association panel size can improve the power of a GWAS, care needs to be taken to ensure that lower LD subpopulations, such as the tropical/subtropical subpopulation, are adequately represented in an association panel in order to capture rare allele variants associated with those subpopulations.

### Candidate genes

We used the B73 maize genome reference sequence to identify genes that either included or were nearby SNPs significantly associated with ear rot resistance. The chromosome 9 gene (GRMZM2G178880) that was identified in both the North Carolina and combined analyses belongs to the cellulose synthase-like family A (*CslA*) protein family. Given that the associated SNP localized to an intron segment within this gene, it is likely that this SNP is in LD with the causal variant and not the causal variant itself. The expression of this gene is highest in the endosperm of the developing seed kernel between 20 and 24 d after flowering during the growing season (Sekhon *et al.* 2011; http://www.plexdb.org). Peak expression of this gene coincides with the initial onset of *Fusarium* ear rot symptoms, which occurs approximately 28 d after flowering (Bush *et al.* 2004). Genes in the *CslA* family encode for noncellulose polysaccharides (such as mannan polymers) that form part of the wall matrix in plant cells (Dhugga 2005; Liepman *et al.* 2005). In the model grass species *Brachypodium distachyon*, mannan polymers make up a significant portion of the seed endosperm (Guillon *et al.* 2011). Dismantling of mannan-rich cell walls may play an important role in programmed cell death in host-pathogen interactions (Gadjev *et al.* 2008; Rodríguez-Gacio *et al.* 2012). Although the interaction between *F. verticillioides* and maize is complex, cell wall structure and programmed cell death may play a role in quantitative resistant to the disease (Chivasa *et al.* 2005).

The SNP on chromosome 5 is located downstream of an HSP60 gene (GRMZM2G111477). Expression levels of this gene are highest in the developing endosperm 12 d after flowering (Sekhon *et al.* 2011; http://www.plexdb.org). HSP60s are chaperonins that are involved with protein folding under plant stress primarily in the mitochondria and cholorplasts (Wang *et al.* 2004). The role of HSP60s in programmed cell death has been demonstrated in mutants of both rice and Arabidopsis (Ishikawa *et al.* 2003; Tsunezuka *et al.* 2005). The SNP on chromosome 1 is contained within the coding region of GRMZM2G703598. Unfortunately, this gene has no predicted function and has no sequence orthology with related grass species.

In conclusion, we used GWAS to identify three novel loci associated with improved resistance to *Fusarium* ear rot in maize. The identified loci each explain a relatively small proportion of the overall phenotypic variance for ear rot, and each locus has a very small additive genetic effect on resistance, consistent with the highly quantitative nature of the *F. verticillioides*-maize pathosystem. The large amount of variation captured by the kinship matrix, in combination with high false discovery rates for the vast majority of SNPs, suggests that additive polygenic variation across many loci underlies resistance to *Fusarium* ear rot. Given the rapid decay of LD along the chromosomes in the maize core diversity panel (Olukolu *et al.* 2013), future studies employing increased marker density and larger association panels may be able to identify other novel loci associated with ear rot resistance. Maize breeders can use targeted allele selection for these three resistance alleles, but may need to also select for recombinations near them as they are introgressed into elite maize from unadapted or undesirable genotypes (such as the tropical maize or popcorn germplasm pools that appear to be enriched for resistance alleles). In addition, given the substantial additive polygenic variation for ear rot resistance, phenotypic and genomic selection approaches should be effective as long as high quality phenotypic evaluations of resistance can be performed to permit direct selection or provide training data for genomic selection models.

## Supplementary Material

Supporting Information
