# Supplementary material for: A Genome-Wide Association Study Reveals Genes Associated with Fusarium Ear Rot Resistance in a Maize Core Diversity Panel
Source: G3 (Bethesda). 2013 Nov 1;3(11):2095–104. doi: 10.1534/g3.113.007328 (PMC3815068; doi:10.1534/g3.113.007328)
Supplement: Supporting Information [file supp_3_11_2095__index.html]

A Genome-Wide Association Study Reveals Genes Associated with Fusarium Ear Rot Resistance in a Maize Core Diversity Panel — Supporting Information 

# A Genome-Wide Association Study Reveals Genes Associated with Fusarium Ear Rot Resistance in a Maize Core Diversity Panel

## Supporting Information for Zila *et al.*, 2013

**Files in this Data Supplement:**

- Supporting Information - Figures S1-S5, Tables S1-S2, and Files S1-S3 (PDF, 1 MB)
- Figure S1 - (A) Example of a susceptible (100% severity) phenotype. (B) Example of a resistant (0% severity) phenotype. (PDF, 359 KB)
- Figure S2 - Scatter plot matrix illustrating the genotypic relationship of Fusarium ear rot resistance between environments. (PDF, 406 KB)
- Figure S3 - Estimating the false discovery rate (FDR) for SNP marker association with Fusarium ear rot resistance in the North Carolina analysis. (PDF, 446 KB)
- Figure S4 - Estimating the false discovery rate (FDR) for SNP marker association with Fusarium ear rot resistance in the Galicia analysis. (PDF, 446 KB)
- Figure S5 - Estimating the false discovery rate (FDR) for SNP marker association with Fusarium ear rot resistance in the combined analysis. (PDF, 446 KB)
- Table S1 - Heritability estimates for Fusarium ear rot resistance, mean ear rot severity, heritability estimates for silking date, regression coefficients for silking date covariates, and significance level of regression coefficients. (PDF, 411 KB)
- Table S2 - Climate data for the three North Carolina and two Galicia environments. (PDF, 307 KB)
- File S1 - Raw phenotypic data from three years in North Carolina and two years in Galicia. (.csv, 415 KB)
- File S2 - Least square means for 267 inbred lines estimated within each experiment (North Carolina and Galicia) and across experiments. Formatted for analysis in Tassel software. (.txt, 7 KB)
- File S3 - A 279 x 279 genetic kinship matrix (**K**) based on Van Raden (2008). Formatted for analysis in Tassel software. (.txt, 953 KB)
