# Supplementary material for: A Genome-Wide Association Study Reveals Genes Associated with Fusarium Ear Rot Resistance in a Maize Core Diversity Panel
Source: G3 (Bethesda). 2013 Nov 1;3(11):2095–104. doi: 10.1534/g3.113.007328 (PMC3815068; doi:10.1534/g3.113.007328)
Supplement: Supporting Information [file supp_g3.113.007328_007328SI.pdf]

G3: Genes | Genomes | Genetics

A Genome-Wide Association Study Reveals Genes Associated with Fusarium Ear Rot Resistance in a Maize Core Diversity Panel

Charles T. Zila[*], L. Fernando Samayoa[§], Rogelio Santiago[§], Ana Butrón[§], and James B. Holland[*†1]

[*]Department of Crop Science, North Carolina State University, Raleigh, North Carolina 27695, [§]Misión Biológica de Galicia, CSIC, Pontevedra, Spain, 36080, and [†]U.S. Department of Agriculture—Agricultural Research Service, Plant Science Research Unit, Raleigh, North Carolina, 27695

[1] Corresponding author: USDA-ARS and Department of Crop Science, Campus Box 7620, Raleigh, NC, 27695-7616. Phone: (919) 513-4198. E-mail: james_holland@ncsu.edu.

DOI: 10.1534/g3.113.007328

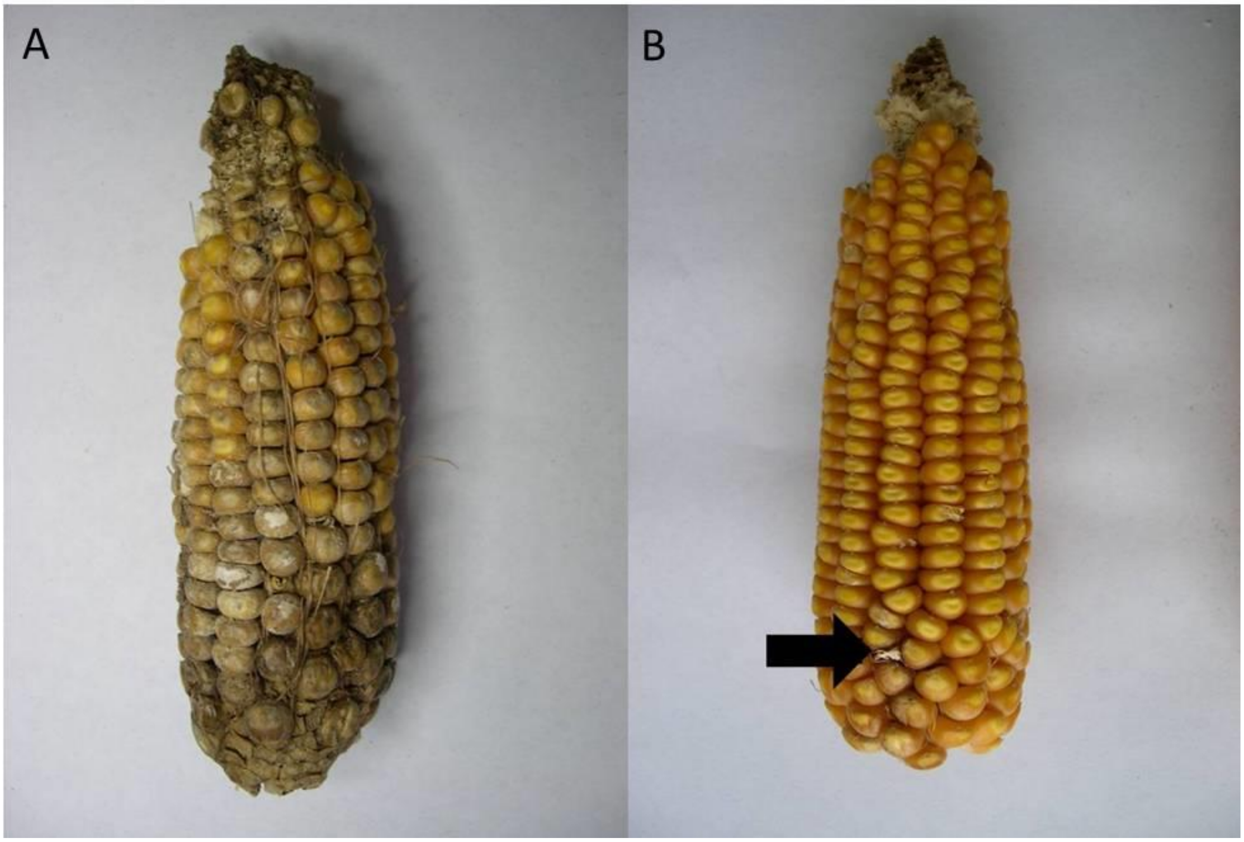

Figure S1 (A) Example of a susceptible (100% severity) phenotype. (B) Example of a resistant (0% severity) phenotype. The arrow indicates the point of inoculation in the resistant ear.

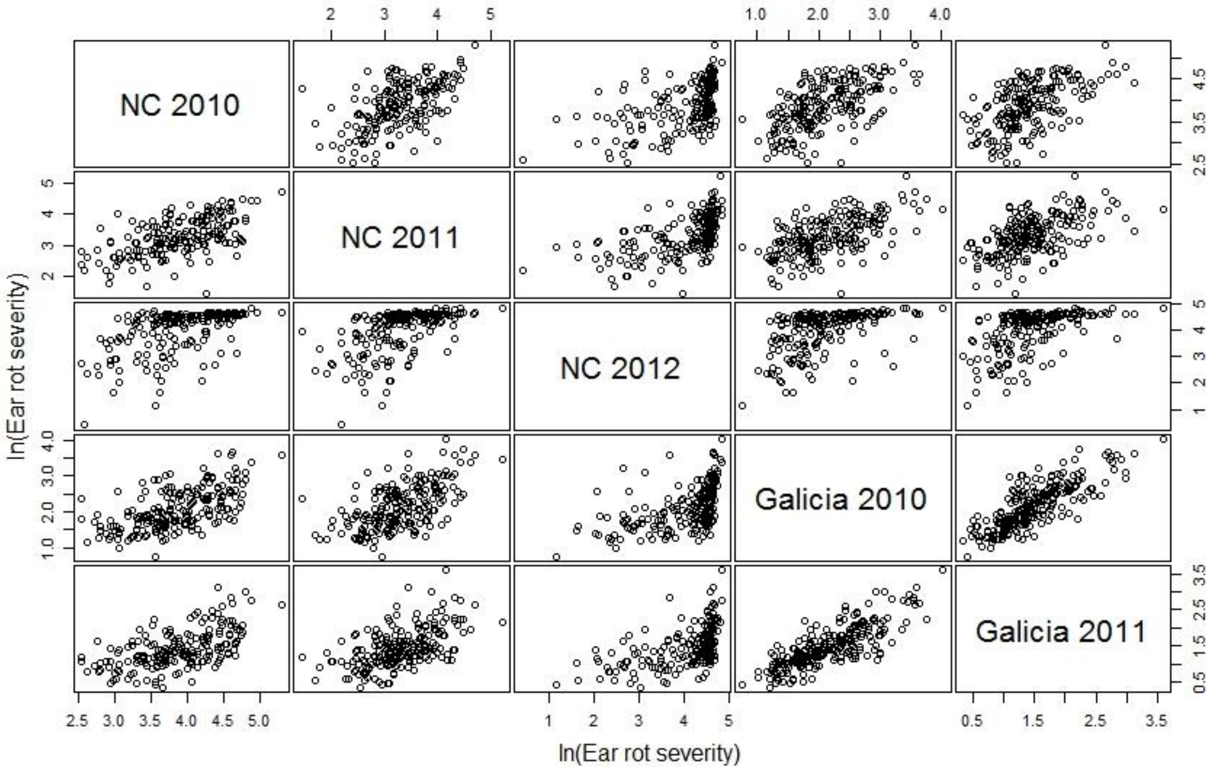

Figure S2 Scatter plot matrix illustrating the genotypic relationship of Fusarium ear rot resistance between environments. The model used to estimate variance components and genetic correlations in the combined analysis was used to predict least square means for each inbred line within each environment (treating line as a fixed effect instead of random). Means for each line on the natural log transformed scale are plotted against one another in each pair-wise combination of environments.

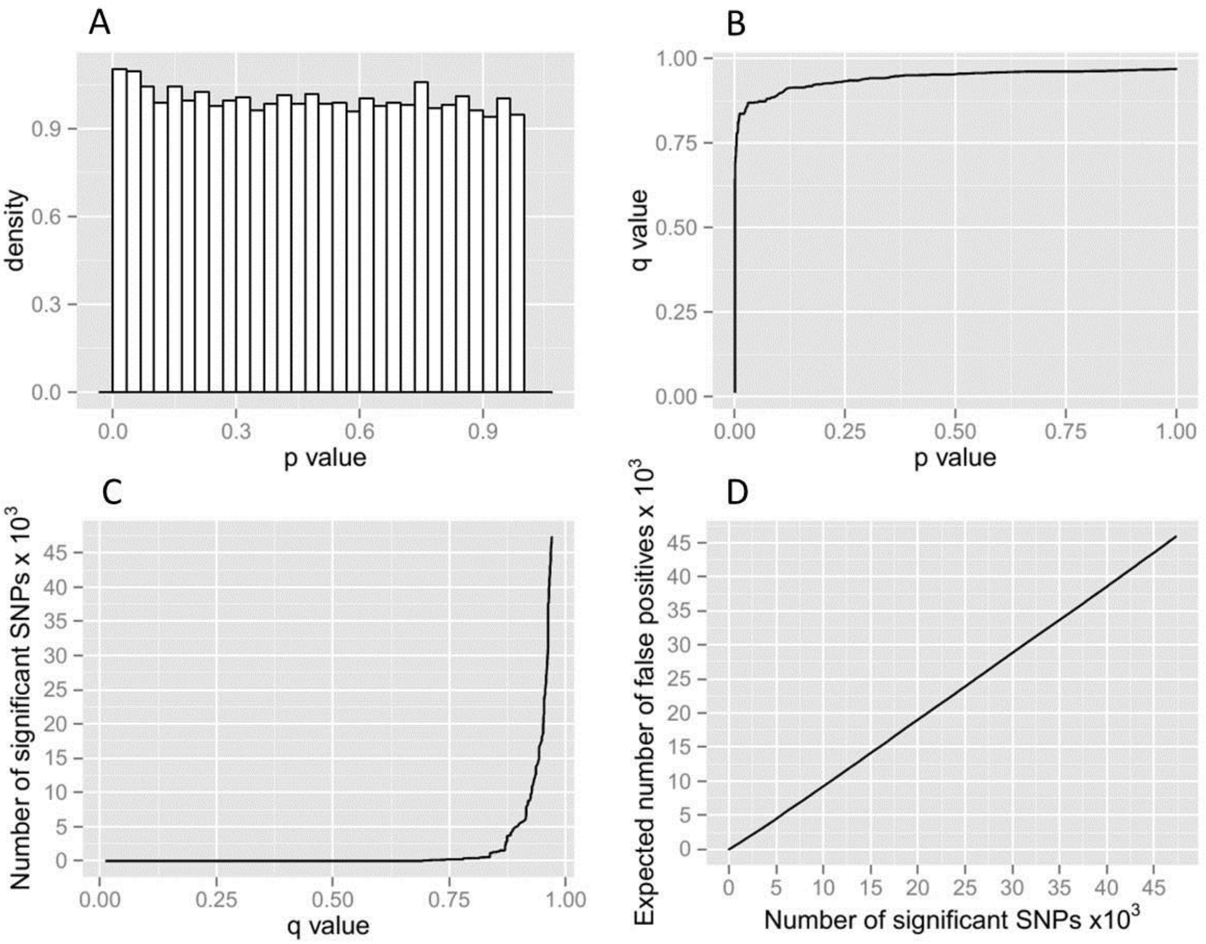

Figure S3 Estimating the false discovery rate (FDR) for SNP marker association with Fusarium ear rot resistance in the North Carolina analysis. (A) A density histogram showing *p*-value distribution of 47,445 SNPs following GWAS. (B) The *q*-values plotted against their respective *p*-values. (C) The number of SNPs plotted against each of the respective *q*-value estimates. (D) The expected number of false positive SNPs versus the total number of significant SNPs given the *q*-values.

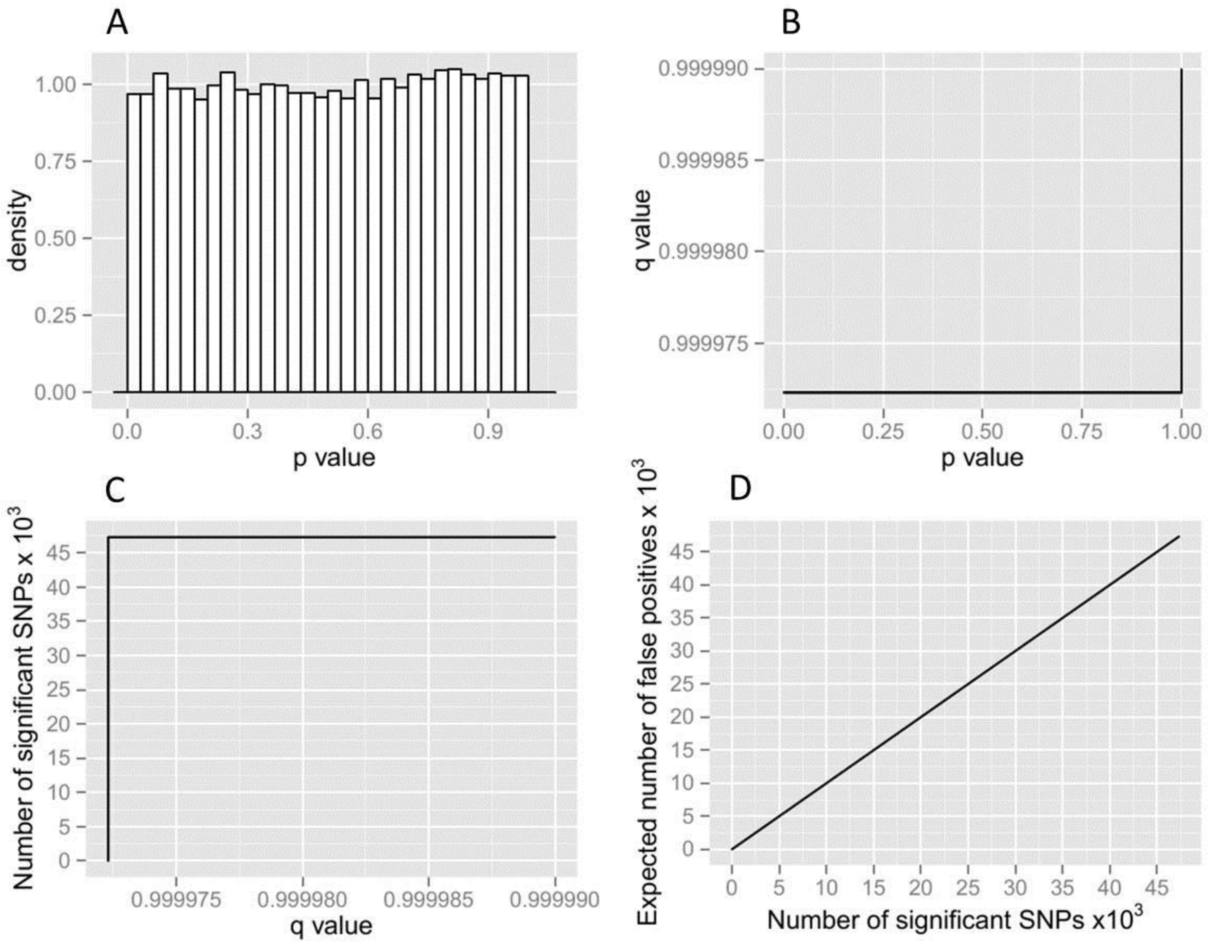

Figure S4 Estimating the false discovery rate (FDR) for SNP marker association with Fusarium ear rot resistance in the Galicia analysis. (A) A density histogram showing *p*-value distribution of 47,445 SNPs following GWAS. (B) The *q*-values plotted against their respective *p*-values. (C) The number of SNPs plotted against each of the respective *q*-value estimates. (D) The expected number of false positive SNPs versus the total number of significant SNPs given the *q*-values.

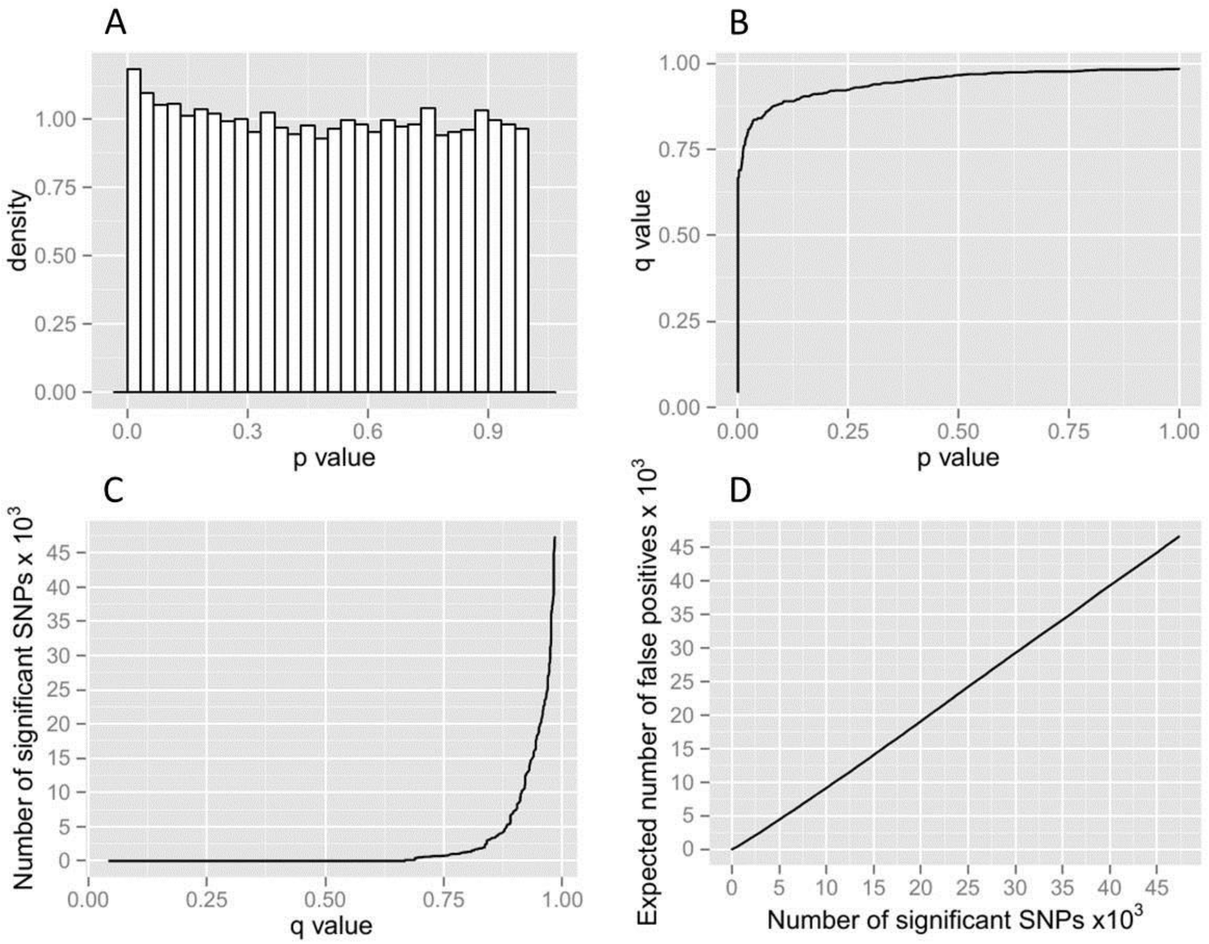

Figure S5 Estimating the false discovery rate (FDR) for SNP marker association with Fusarium ear rot resistance in the combined analysis. (A) A density histogram showing *p*-value distribution of 47,445 SNPs following GWAS. (B) The *q*-values plotted against their respective *p*-values. (C) The number of SNPs plotted against each of the respective *q*-value estimates. (D) The expected number of false positive SNPs versus the total number of significant SNPs given the *q*-values.

Table S1 Heritability estimates for Fusarium ear rot resistance, mean ear rot severity, heritability estimates for silking date, regression coefficients for silking date covariates, and significance level of regression coefficients. Estimates are reported for each environment individually, across years within the North Carolina and Galicia environments, and combined across all environments.

	Fusarium ear rot		Silking date		
Environment	$\widehat{H}_c$	Mean (%)[a]	$\widehat{H}_c$	$\hat{\beta}$ (%/day)[b]	*P*-value
NC 2010	0.44	46.7	0.92	0.03	0.023
NC 2011	0.47	26.8	0.38	0.04	0.024
NC 2012	0.71	55.1	0.78	0.01	0.340
Galicia 2010	0.53	7.6	0.93	0.05	0.022
Galicia 2011	0.51	3.4	0.90	0.02	0.248
NC, all years	0.73	41.1	0.95	0.02	<0.001
Galicia, all years	0.71	7.4	0.92	0.02	0.099
Combined	0.75	22.1	0.98	0.02	<0.001

[a] Mean ear rot severity is reported as the average of the line least square means calculated within and across environments. Means are reported back-transformed to the original 0-100% disease severity scale.

[b] Regression coefficients for the silking date covariate in the Fusarium ear rot models. Coefficients are reported back-transformed to the original 0-100% disease severity scale. As an example, a one day increase in silking date in NC 2010 increased the ear rot score for an observation by 0.03%.

Table S2 Climate data for the three North Carolina and two Galicia environments. Average daily minimum temperature, average daily maximum temperature, average daily overall temperature, and cumulative precipitation level are reported for two time intervals in each environment: planting date to the average silking date (date at which at least 50% of the plots within an environment had silked) and average silking date to 45 days post-silking.

Environment	Planting date to average silking date				Average silking date to 45 days post-silking			
	Average daily min. temp. (°C)	Average daily max. temp. (°C)	Average daily temp. (°C)	Cumulative precipita-tion (mm)	Average daily min. temp. (°C)	Average daily max. temp. (°C)	Average daily temp. (°C)	Cumulative precipita-tion (mm)
NC 2010	17.5	28.4	23.0	252.7	21.9	32.7	27.3	120.7
NC 2011	17.4	28.9	23.1	70.6	22.1	33.5	27.8	90.9
NC 2012	16.1	27.3	21.7	9.1	22.3	32.1	27.2	258.8
Galicia 2010	7.0	19.5	13.3	356.2	13.3	25.6	19.5	81.3
Galicia 2011	10.8	25.8	19.7	63.1	11.4	26.5	18.7	137.2

Files S1-S3

Supporting data

Available for download at http://www.g3journal.org/lookup/suppl/doi:10.1534/g3.113.007328/-/DC1

File S1 Raw phenotypic data from three years in North Carolina and two years in Galicia. Formatted for analysis in ASReml software. Columns in the data file are as follows from left to right: location (Loc, 1=NC, 2=Galicia), environment (Env, a unique combination of location and year), year, row (field position of plot from front of the field to the back), column (field position of plot from left to right), set ("99" is a placeholder in Galicia), rep ("99" is a placeholder in North Carolina), block, plot number, line name (Material), entry number, flowering date (converted to the number of days after planting until flowering), Fusarium ear rot score (rot_AVG, averaged across ears within the plot), number of ears scored within each plot (earno), and the natural log transformation of the average ear rot score (logrot).

File S2 Least square means for 267 inbred lines estimated within each experiment (North Carolina and Galicia) and across experiments. Formatted for analysis in Tassel software. Column <trait> (Tassel nomenclature) contains the line names corresponding to File S1, and the other columns are as follows: least square means based on North Carolina data (NC_BLUE), means based on Galicia data (ES_BLUE), and means based on combined data (Overall_BLUE).

File S3 A 279 ×279 genetic kinship matrix (**K**) based on Van Raden (2008). Formatted for analysis in Tassel software. The first column contains line names, and all other columns contain the pair-wise kinship coefficients between lines.
