# Supplementary material for: A Genome-Wide Association Study Reveals Genes Associated with Fusarium Ear Rot Resistance in a Maize Core Diversity Panel
Source: G3 (Bethesda). 2013 Nov 1;3(11):2095–104. doi: 10.1534/g3.113.007328 (PMC3815068; doi:10.1534/g3.113.007328)
Supplement: Supporting Information [file supp_g3.113.007328_FigureS1.pdf]

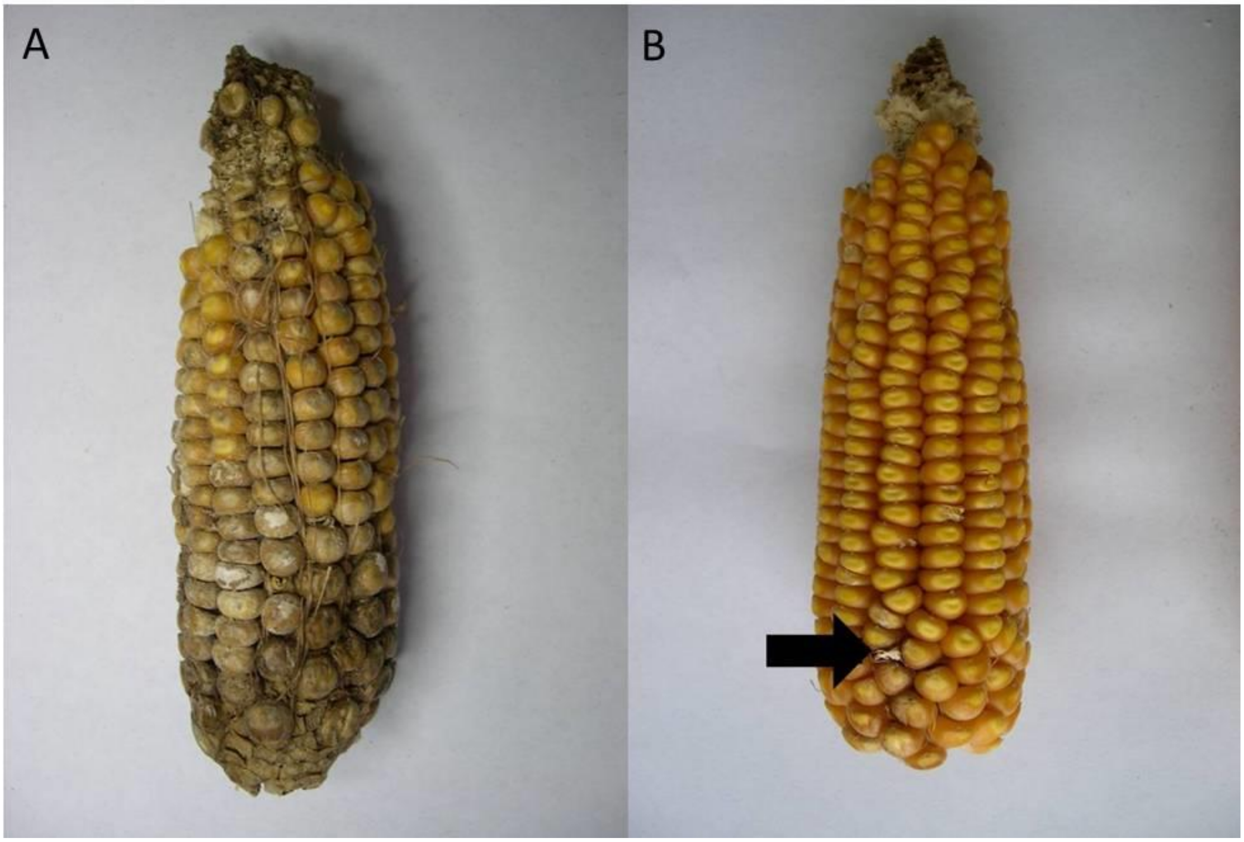

Figure S1 (A) Example of a susceptible (100% severity) phenotype. (B) Example of a resistant (0% severity) phenotype. The arrow indicates the point of inoculation in the resistant ear.
