# Supplementary material for: A Genome-Wide Association Study Reveals Genes Associated with Fusarium Ear Rot Resistance in a Maize Core Diversity Panel
Source: G3 (Bethesda). 2013 Nov 1;3(11):2095–104. doi: 10.1534/g3.113.007328 (PMC3815068; doi:10.1534/g3.113.007328)
Supplement: Supporting Information [file supp_g3.113.007328_FigureS2.pdf]

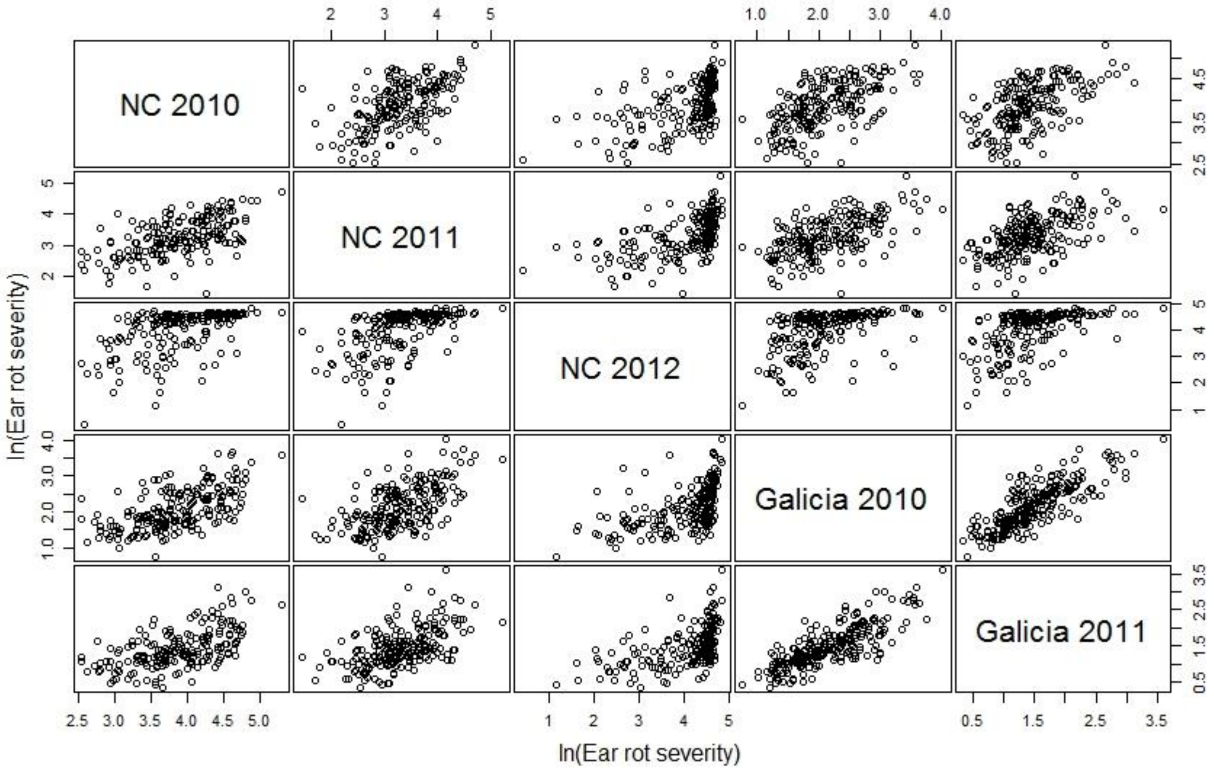

Figure S2 Scatter plot matrix illustrating the genotypic relationship of Fusarium ear rot resistance between environments. The model used to estimate variance components and genetic correlations in the combined analysis was used to predict least square means for each inbred line within each environment (treating line as a fixed effect instead of random). Means for each line on the natural log transformed scale are plotted against one another in each pair-wise combination of environments.
