# Supplementary material for: A Genome-Wide Association Study Reveals Genes Associated with Fusarium Ear Rot Resistance in a Maize Core Diversity Panel
Source: G3 (Bethesda). 2013 Nov 1;3(11):2095–104. doi: 10.1534/g3.113.007328 (PMC3815068; doi:10.1534/g3.113.007328)
Supplement: Supporting Information [file supp_g3.113.007328_FigureS3.pdf]

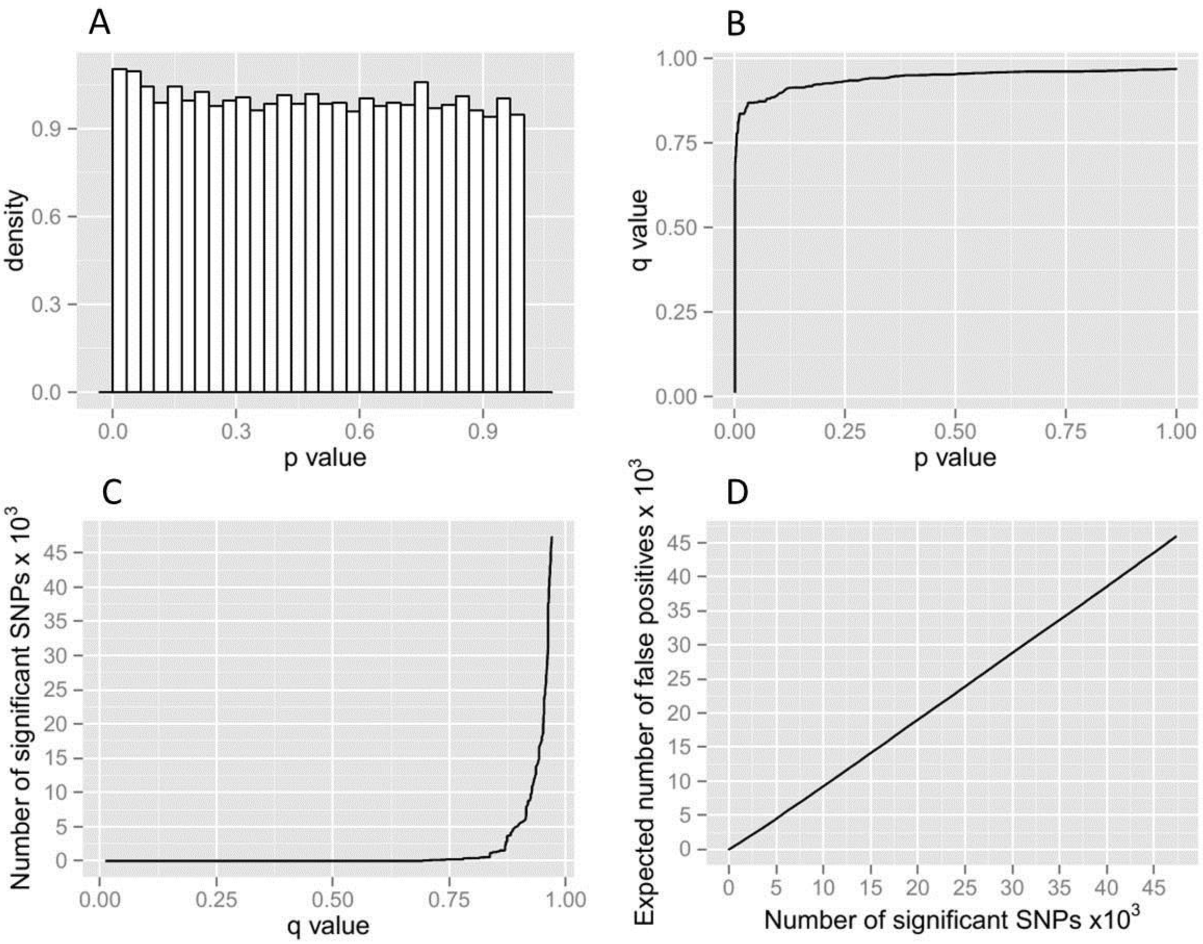


**Figure S3** Estimating the false discovery rate (FDR) for SNP marker association with Fusarium ear rot resistance in the North Carolina analysis. (A) A density histogram showing *p*-value distribution of 47,445 SNPs following GWAS. (B) The *q*-values plotted against their respective *p*-values. (C) The number of SNPs plotted against each of the respective *q*-value estimates. (D) The expected number of false positive SNPs versus the total number of significant SNPs given the *q*-values.
