# Supplementary material for: A Genome-Wide Association Study Reveals Genes Associated with Fusarium Ear Rot Resistance in a Maize Core Diversity Panel
Source: G3 (Bethesda). 2013 Nov 1;3(11):2095–104. doi: 10.1534/g3.113.007328 (PMC3815068; doi:10.1534/g3.113.007328)
Supplement: Supporting Information [file supp_g3.113.007328_TableS1.pdf]

Table S1 Heritability estimates for Fusarium ear rot resistance, mean ear rot severity, heritability estimates for silking date, regression coefficients for silking date covariates, and significance level of regression coefficients. Estimates are reported for each environment individually, across years within the North Carolina and Galicia environments, and combined across all environments.

	Fusarium ear rot		Silking date		
Environment	$\widehat{H}_c$	Mean (%)[a]	$\widehat{H}_c$	$\hat{\beta}$ (%/day)[b]	*P*-value
NC 2010	0.44	46.7	0.92	0.03	0.023
NC 2011	0.47	26.8	0.38	0.04	0.024
NC 2012	0.71	55.1	0.78	0.01	0.340
Galicia 2010	0.53	7.6	0.93	0.05	0.022
Galicia 2011	0.51	3.4	0.90	0.02	0.248
NC, all years	0.73	41.1	0.95	0.02	<0.001
Galicia, all years	0.71	7.4	0.92	0.02	0.099
Combined	0.75	22.1	0.98	0.02	<0.001

[a] Mean ear rot severity is reported as the average of the line least square means calculated within and across environments. Means are reported back-transformed to the original 0-100% disease severity scale.

[b] Regression coefficients for the silking date covariate in the Fusarium ear rot models. Coefficients are reported back-transformed to the original 0-100% disease severity scale. As an example, a one day increase in silking date in NC 2010 increased the ear rot score for an observation by 0.03%.
