# Supplementary material for: A Genome-Wide Association Study Reveals Genes Associated with Fusarium Ear Rot Resistance in a Maize Core Diversity Panel
Source: G3 (Bethesda). 2013 Nov 1;3(11):2095–104. doi: 10.1534/g3.113.007328 (PMC3815068; doi:10.1534/g3.113.007328)
Supplement: Supporting Information [file supp_g3.113.007328_TableS2.pdf]

Table S2 Climate data for the three North Carolina and two Galicia environments. Average daily minimum temperature, average daily maximum temperature, average daily overall temperature, and cumulative precipitation level are reported for two time intervals in each environment: planting date to the average silking date (date at which at least 50% of the plots within an environment had silked) and average silking date to 45 days post-silking.

	Planting date to average silking date				Average silking date to 45 days post-silking			
Environment	Average daily min. temp. (°C)	Average daily max. temp. (°C)	Average daily temp. (°C)	Cumulative precipita-tion (mm)	Average daily min. temp. (°C)	Average daily max. temp. (°C)	Average daily temp. (°C)	Cumulative precipita-tion (mm)
NC 2010	17.5	28.4	23.0	252.7	21.9	32.7	27.3	120.7
NC 2011	17.4	28.9	23.1	70.6	22.1	33.5	27.8	90.9
NC 2012	16.1	27.3	21.7	9.1	22.3	32.1	27.2	258.8
Galicia 2010	7.0	19.5	13.3	356.2	13.3	25.6	19.5	81.3
Galicia 2011	10.8	25.8	19.7	63.1	11.4	26.5	18.7	137.2
